# Canagliflozin Improves Erythropoiesis in Diabetes Patients with Anemia of Chronic Kidney Disease

**DOI:** 10.1089/dia.2019.0212

**Published:** 2019-11-22

**Authors:** Takashi Maruyama, Hiroyuki Takashima, Hidetaka Oguma, Yoshihiro Nakamura, Michiko Ohno, Kei Utsunomiya, Tetsuya Furukawa, Ritsukou Tei, Masanori Abe

**Affiliations:** Division of Nephrology, Hypertension and Endocrinology, Department of Internal Medicine, Nihon University School of Medicine, Tokyo, Japan.

**Keywords:** Canagliflozin, Chronic kidney disease, Erythropoiesis, Erythropoietin, Renal anemia, Type 2 diabetes

## Abstract

***Background:*** We evaluated the erythropoietic effects of canagliflozin, a sodium-glucose cotransporter 2 inhibitor, in type 2 diabetes patients with anemia of chronic kidney disease.

***Methods:*** Nine diabetes patients were enrolled and administered 100 mg canagliflozin once a day for 12 weeks. The patients received fixed doses of conventional antidiabetic drugs and renin-angiotensin system inhibitors for 8 weeks before enrollment; these drugs were continued during the study. Endpoints were changes in erythropoiesis parameters, including erythrocyte and reticulocyte count, hemoglobin, hematocrit, and serum erythropoietin (EPO) concentration from baseline to 12 weeks. All variables were measured every 2 weeks.

***Results:*** Serum EPO concentration increased by 38 [15–62]% (*P* = 0.043) between baseline and 2 and 4 weeks. Reticulocyte count transiently increased at 2 weeks. Erythropoiesis occurred after 2 weeks of canagliflozin treatment. Erythrocyte count (from 386 ± 36 × 10^4^/μL to 421 ± 36 × 10^4^/μL; *P* = 0.0009), hemoglobin (from 11.8 ± 0.6 g/dL to 12.9 ± 1.1 g/dL; *P* = 0.0049), and hematocrit (from 37.1 ± 2.3% to 40.4 ± 3.2%; *P* = 0.002) increased from baseline to study completion. Although there were no significant changes in transferrin saturation, serum ferritin levels were decreased (*P* = 0.003).

***Conclusions:*** Canagliflozin treatment led to an improvement in erythropoiesis in patients with impaired kidney function. The effect on erythropoiesis appeared to be due to an EPO production-mediated mechanism and might be independent of glycemic control; however, further studies are needed to clarify this since the present study had a small sample size and no comparator group.

## Introduction

Sodium-glucose cotransporter 2 (SGLT2) inhibitors increase the excretion of glucose into the urine by inhibiting urinary glucose reabsorption in the proximal tubule, which leads to reduced blood glucose levels, body weight, and blood pressure.^1,2^ In addition, SGLT2 inhibitors are expected to have renoprotective effects based on the possible mechanism that they reduce hyperfiltration by blocking proximal tubule sodium reabsorption and activating tubuloglomerular feedback,^3–5^ and may decrease the inflammatory, fibrotic, and hyperplastic responses of proximal tubular cells by blocking glucose reabsorption into proximal tubular cells.^6^

Hemoglobin levels increase soon after the initiation of SGLT2 inhibitor therapy, and remain elevated as long as SGLT2 inhibitor administration continues.^7^ Although a decrease in plasma volume, with resultant hemoconcentration, could contribute to this increase in hemoglobin, it seems that changes in hemoglobin during SGLT2 inhibitor administration are largely dependent on factors other than hemoconcentration. Erythropoietin (EPO) is produced by renal interstitial fibroblasts, and the regulation of EPO production by interstitial fibroblasts involves oxygen sensing through hypoxia-inducible factor (HIF).^8^ In patients with diabetes, excessive glucose reabsorption overtaxes the proximal tubules, which increases the oxygen requirement and causes tubulointerstitial hypoxia. As a consequence, damaged renal tubules undergo transformation into dysfunctional fibroblasts, which might impair EPO production. SGLT2 inhibitors might reduce the workload of the proximal tubules and improve tubulointerstitial hypoxia, allowing fibroblasts to resume EPO production. The mechanism underlying this elevation of hemoglobin is presumed to involve the enhancement of erythropoiesis in patients with normal kidney function.^9^ However, the effects of SGLT2 inhibition are unclear with regard to erythropoiesis in patients with chronic kidney disease. In the present study, we aimed to examine precisely the effects of the SGLT2 inhibitor canagliflozin on erythropoiesis by measuring erythropoiesis parameters, including serum EPO concentration, kidney function parameters, and other metabolic parameters in type 2 diabetes mellitus patients with renal anemia, who are treated with renin-angiotensin system inhibitors.

## Materials and Methods

### Subjects

The enrollment criteria were as follows: (1) type 2 diabetes mellitus with nephropathy under stable glycemic control, defined as a hemoglobin A1c (HbA1c) level <10.0% after the administration of oral antidiabetic agents, insulin injection therapy, or diet therapy alone for eight consecutive weeks; (2) albuminuria: urinary albumin-to-creatinine ratio (UACR) of 30–3000 mg/g creatinine (average of two consecutive measurements recorded during the 8-week pretreatment period); (3) estimated glomerular filtration rate (eGFR) of 45–60 mL/min/1.73 m^2^; (4) anemia of chronic kidney disease defined as hemoglobin <12.5 g/dL (average of two consecutive measurements recorded during the 8-week pretreatment period) without iron deficiency defined as serum ferritin levels >50 ng/mL^10,11^; and (5) hypertension treated with a fixed dose of renin-angiotensin system inhibitors for at least 8 weeks before the study treatment began. The exclusion criteria were as follows: (1) age <20 or >85 years; (2) eGFR <45 or ≥60 mL/min/1.73 m^2^; (3) HbA1c ≥10.0%; (4) severe heart failure, angina, myocardial infarction, or stroke occurring within 6 months before enrollment in the trial; and (5) treatment with an SGLT2 inhibitor and/or erythropoiesis-stimulating agent within 8 weeks of enrollment in the trial.

### Study design and treatments

This prospective, open-label, single-arm, single-center study was conducted between October 2018 and March 2019, and eligible patients received oral canagliflozin (100 mg/day) for 12 weeks. Before enrollment, the patients received fixed doses of conventional antidiabetic drugs (oral hypoglycemic agents and/or insulin) for 8 weeks, and these drugs were continued during the 12-week treatment period. If the investigator believed that canagliflozin presented a safety problem, its administration was discontinued. The patients continued their regular medications, such as antihypertensive drugs and lipid-lowering agents, during the study period as long as the investigators were able to adjust antihypertensive drugs during the trial to improve blood pressure control. The study protocol was approved by the Ethics Committee of Nihon University Itabashi Hospital, and all patients provided written informed consent (Clinical Trial Registration No. UMIN0000 36183). The study protocol was designed in accordance with the Declaration of Helsinki.

### Study evaluations and endpoints

The primary efficacy endpoint was a change in erythropoiesis, as measured by erythrocyte count, hemoglobin, hematocrit, reticulocyte count, and EPO concentration from baseline to 12 weeks. All variables were measured every 2 weeks during the 12-week study period.

The secondary endpoints included changes in laboratory/biochemical tests and vital signs during the study, and safety. Serum creatinine level and eGFR were measured at baseline and at weeks 4, 8, and 12. Serum ferritin levels and transferrin saturation (TSAT) were measured at baseline and at weeks 4, 8, and 12. TSAT was calculated according to the following formula: TSAT (%) = serum iron/total iron binding capacity × 100. Serum vitamin B_12_, folic acid, and zinc levels were measured at baseline and at the end of the study. HbA1c and casual plasma glucose levels were measured as indices of glycemic control. Casual plasma glucose was defined at the discretion of each investigator. Vital signs, including body weight, body mass index, and systolic and diastolic blood pressure, were recorded at each visit. Aspartate aminotransferase, alanine aminotransferase, γ-glutamyl transpeptidase, total cholesterol, high-density lipoprotein cholesterol, and triglyceride levels were measured by routine clinical chemistry procedures using commercially available assay kits. The serum concentration of low-density lipoprotein cholesterol was estimated using the Friedewald formula (low-density lipoprotein cholesterol = total cholesterol − high-density lipoprotein cholesterol − triglyceride × 0.2) in patients with serum triglyceride concentrations <400 mg/dL.^12^ eGFR was calculated according to the following formula for Japanese patients: eGFR (mL/min/1.73 m^2^) = 194 × serum creatinine^−1.094^ × age^−0.287^ ( × 0.739 for women).^13^

Albuminuria was assessed by measuring the UACR in the first urine specimen of the day. Urinary albumin levels were measured by an immunoturbidimetric assay. Urinary liver-type fatty acid binding protein (L-FABP) was measured by a specific chemiluminescent enzyme immunoassay (SRL, Inc., Tokyo, Japan) in the same urine sample, and the values were expressed relative to the urinary creatinine concentration. Urinary *N*-acetyl-β-d-glucosaminidase (NAG) and β_2_-microglobulin (β_2_MG) concentrations in the same urine sample were measured by colorimetry and latex agglutination, respectively.

All variables were assessed at baseline and at the end of the study. Efficacy variables were analyzed in all subjects. The subjects could be withdrawn in the event of allergy or intolerance to the drug, if either serum transaminase concentration or creatine kinase concentration increased to more than 2 × the upper limit of normal, or following an event that, in the investigator's opinion, might have posed a risk to the patient or confounded the results of the study. At each visit, the subjects were questioned about study compliance (diet and medications), concomitant medications, and adverse events. Safety assessments were performed throughout the study. Adverse events were graded by intensity: mild, moderate, or severe. Serious adverse events were defined as medical events that resulted in death, hospitalization, or significant disability or incapacity.

### Statistical analysis

Data are expressed as the mean ± standard deviation or median (interquartile range) as appropriate. Changes from baseline were analyzed using two-tailed paired *t*-tests. Associations between the changes of EPO concentration at each time point and other hematopoiesis variables were assessed using Spearman's correlation coefficients. Statistical significance was set at *P* < 0.05. All analyses were performed using JMP software version 12 (SAS Institute Ltd., Cary, NC).

## Results

### Subjects

A total of nine subjects (seven men and two women) were treated with 100 mg canagliflozin for 12 weeks. None of the subjects withdrew after consenting to participate in the study. At baseline, the subjects had an HbA1c level of 6.7 ± 0.6% and were 71 ± 14 years old. Mean body weight was 63.6 ± 7.7 kg, and mean body mass index was 24.3 ± 3.3 kg/m^2^. One patient had a history of cardiovascular disease. Systolic and diastolic blood pressures at baseline were 143 ± 13 and 79 ± 13 mmHg, respectively. eGFR and UACR at baseline were 45.2 ± 6.2 mL/min/1.73m^2^ and 191 (68–2255) mg/g creatinine, respectively (Table 1). All patients were taking an angiotensin II receptor blocker. Data were available for all nine patients at baseline and at the end of the study. The canagliflozin dose was not changed in any patient, and none of the patients started taking another antidiabetic drug, antihypertensive drug, erythropoiesis-stimulating agent, or iron supplementation during the study period.

**Table 1.** Characteristics and Medications of the Study Patients at Baseline

*Variable*	
*n* (male/female)	9 (7/2)
Age, years	71 ± 14
eGFR, mL/min/1.73 m^2^	45.2 ± 6.2
UACR, mg/g Cr	191 (68–2255)
HbA1c, %	6.7 ± 0.6
Medications	
Antidiabetic agents, *n* (%)	
DPP-4 inhibitors	6 (66.7)
Insulin	2 (22.2)
α-Glucosidase inhibitors	1 (11.1)
Metformin	0 (0)
Sulfonylurea	0 (0)
Antihypertensive agents, *n* (%)	
Angiotensin II receptor blockers	9 (100)
ACE inhibitors	0 (0)
Mineralocorticoid receptor antagonist	0 (0)
Calcium channel blockers	7 (77.8)
Diuretics	1 (11.1)
α-Blockers	2 (22.2)
β-Blockers	2 (22.2)
Antihyperuricemic agents, *n* (%)	5 (55.6)
Statins, *n* (%)	5 (55.6)

Data are expressed as mean ± standard deviation or median (interquartile range).

ACE, angiotensin converting enzyme; Cr, creatinine; DPP-4, dipeptidyl peptidase-4; eGFR, estimated glomerular filtration rate; HbA1c, hemoglobin A1c; UACR, urinary albumin-to-creatinine ratio.

### Primary endpoint

**FIG. 1.**
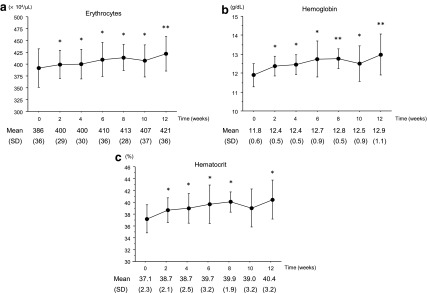
Change in erythropoiesis profiles. **(a)** Changes in erythrocyte count at each time point. Data are expressed as mean ± SD. **(b)** Changes in hemoglobin level at each time point. Data are expressed as mean ± SD. **(c)** Changes in hematocrit level at each time point. Data are expressed as mean ± SD. *P < 0.05; **P < 0.01 versus baseline. SD, standard deviation.

Figure 1 shows the changes in erythropoiesis parameters (erythrocytes, hemoglobin, and hematocrit) during the 12-week treatment period. Erythrocyte counts increased from week 2 until the end of the study period (from 386 ± 36 × 10^4^/μL at baseline to 421 ± 36 × 10^4^/μL at week 12; *P* = 0.0009). Hemoglobin levels also increased from week 2 until the end of the study period (from 11.8 ± 0.6 g/dL at baseline to 12.9 ± 1.1 g/dL at week 12; *P* = 0.0049). Hematocrit levels also increased from week 2 until the end of the study period (from 37.1 ± 2.3% at baseline to 40.4 ± 3.2% at week 12; *P* = 0.002).

**FIG. 2.**
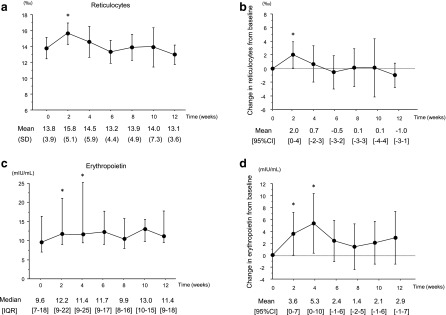
Changes in reticulocyte count and serum erythropoietin concentration. **(a)** Changes in reticulocyte count at each time point. Data are expressed as mean ± SD. **(b)** Changes in reticulocyte count from baseline. Data are expressed as mean and 95% CI. Error bars indicate the 95% CI. **(c)** Changes in serum erythropoietin concentration at each time point. Data are expressed as median and IQR. **(d)** Changes in serum erythropoietin concentration from baseline. Data are expressed as mean and 95% CI. Error bars indicate the 95% CI. *P < 0.05 versus baseline. CI, confidence interval; IQR, interquartile range.

As shown in Figure 2, reticulocyte count was increased between baseline and 2 weeks (*P* = 0.043). Serum EPO concentration was increased (by 38 [15–62]%; *P* = 0.043) between baseline and 4 weeks. Although there were no significant changes in TSAT, serum ferritin levels were decreased (*P* = 0.003). There were no significant changes in serum vitamin B_12_, folic acid, and zinc levels (Table 2).

**Table 2.** Changes in Anemia-Related Parameters

*Variable*	*Baseline*	*End*	P
TSAT, %	30 ± 16	23 ± 8	0.301
Ferritin, ng/mL	72 (55–137)	55 (29–109)	0.003
Vitamin B_12_, pg/mL	470 (293–863)	443 (248–636)	0.149
Folic acid, ng/mL	7.1 ± 1.9	7.1 ± 2.2	0.970
Zinc, μg/dL	61 ± 11	66 ± 12	0.156

Data are expressed as mean ± standard deviation or median (interquartile range).

TSAT, transferrin saturation

### Correlation between EPO concentration and other erythropoietic parameters

The change in serum EPO concentration at each time point was significantly correlated with changes in hemoglobin and hematocrit levels after 2 weeks; the change in EPO concentration from week 2 to 4 was associated with hemoglobin and hematocrit levels at week 6 (*ρ* = 0.446; *P* = 0.004 and *ρ* = 0.369; *P* = 0.019, respectively; Table 3). There were no other significant correlations between the change in EPO concentration and other parameters, including erythrocytes, reticulocytes, hemoglobin, and hematocrit at the same time point.

**Table 3.** Correlation Analysis Between Changes in Serum Erythropoietin Concentration and Other Hematopoiesis Parameters

*Variable*	*Spearman's correlation coefficient*	P
Erythrocytes	0.104	0.520
Hemoglobin	0.093	0.565
Hematocrit	0.208	0.195
Reticulocytes	0.091	0.576
Erythrocytes after 2 weeks	0.172	0.288
Hemoglobin after 2 weeks	0.446	0.004
Hematocrit after 2 weeks	0.369	0.019
Reticulocytes after 2 weeks	0.007	0.961

### Effects of kidney parameters

There were no significant changes in serum creatinine and eGFR levels between baseline and the end of the study. Decreases in eGFR from baseline were observed from week 4 to 6 (from 45.2 ± 6.2 mL/min/1.73m^2^ at baseline to 43.2 ± 6.6 mL/min/1.73m^2^ at week 4 and 42.7 ± 6.3 mL/min/1.73m^2^ at week 6, *P* = 0.017 and 0.003, respectively). The largest reduction in eGFR with canagliflozin administration was observed at week 6, but then tended to increase toward baseline for the remainder of the 12-week treatment period. There were no significant associations between the changes in eGFR and changes in hemoglobin and EPO concentrations. The UACR decreased from 191 (68–2255) mg/g creatinine at baseline to 68 (37–1700) mg/g creatinine at week 12 (*P* = 0.039). There were no significant changes in urinary L-FABP, NAG, and β_2_MG levels (Table 4). Although serum urea nitrogen was significantly increased, uric acid levels were significantly decreased.

**Table 4.** Changes in Vital Signs and Laboratory Variables

*Variable*	*Baseline*	*End*	P
Body mass index, kg/m^2^	24.3 ± 3.3	23.8 ± 3.1	0.012
Systolic BP, mmHg	143 ± 13	135 ± 14	<0.0001
Diastolic BP, mmHg	79 ± 13	75 ± 14	0.024
Heart rate, bpm	78 ± 18	75 ± 18	0.115
Serum creatinine, mg/dL	1.22 ± 0.18	1.24 ± 0.26	0.647
eGFR, mL/min/1.73 m^2^	45.2 ± 6.2	45.2 ± 7.0	0.957
Serum urea nitrogen, mg/dL	18.8 ± 6.5	22.2 ± 6.2	0.002
Uric acid, mg/dL	6.0 ± 1.2	5.5 ± 1.3	0.009
UACR, mg/g Cr	191 (68–2255)	68 (37–1700)	0.039
Urinary NAG, IU/L	13.5 (4.5–22.6)	6.2 (4.0–15.6)	0.628
Urinary β_2_-microglobulin, μg/L	223 (152–1128)	503 (210–1870)	0.912
Urinary L-FABP, μg/g Cr	10.9 (3.2–20.8)	5.8 (2.3–26.4)	0.435
HbA1c, %	6.7 ± 0.6	6.4 ± 0.5	0.014
Casual plasma glucose, mg/dL	140 ± 25	128 ± 18	0.027
Serum albumin, g/dL	3.8 ± 0.7	4.0 ± 0.8	0.036
Total cholesterol, mg/dL	185 ± 35	185 ± 40	0.982
LDL-cholesterol, mg/dL	95 ± 30	91 ± 30	0.285
HDL-cholesterol, mg/dL	46 ± 12	50 ± 13	0.171
Triglycerides, mg/dL	186 (89–335)	160 (108–298)	0.176
AST, U/L	25 ± 7	20 ± 4	0.016
ALT, U/L	21 ± 9	18 ± 8	0.015
γ-GTP, U/L	35 ± 6	30 ± 4	0.0003

Data are expressed as mean ± standard deviation or median (interquartile range).

ALT, alanine aminotransferase; AST, aspartate aminotransferase; BP, blood pressure; Cr, creatinine; eGFR, estimated glomerular filtration rate; γ-GTP, γ-glutamyl transpeptidase; HDL, high-density lipoprotein; L-FABP, liver-type fatty acid binding protein; LDL, low-density lipoprotein; NAG, *N*-acetyl-β-d-glucosaminidase.

### Other laboratory variables and vital signs

Table 4 shows the vital signs and laboratory variables at baseline and at the end of the study. Of note, significant decreases in body mass index and systolic and diastolic blood pressure were observed with canagliflozin treatment. HbA1c (%) decreased from 6.7 ± 0.6 at baseline to 6.4 ± 0.5 at week 12 (*P* = 0.014). The casual plasma glucose level also decreased significantly from baseline. However, serum lipid levels did not change significantly over time. Aspartate aminotransferase, alanine aminotransferase, and γ-glutamyl transpeptidase levels were significantly decreased by canagliflozin treatment.

### Safety

None of the patients exhibited significant adverse events such as symptomatic hypoglycemia, urinary tract infection, genital infection, or limb amputation. Canagliflozin was well tolerated and was not discontinued in any of the patients.

## Discussion

In this study, canagliflozin was found to increase erythrocytes, hemoglobin, and hematocrit levels after 12 weeks of treatment. Furthermore, serum EPO concentration was significantly increased after 2 and 4 weeks of canagliflozin treatment. A similar magnitude of erythropoiesis has been reported with SGLT2 inhibitors in a subpopulation of diabetes patients with normal kidney function.^9,14^ However, our findings are the first, to our knowledge, to show that canagliflozin improves erythropoiesis, including hemoglobin and hematocrit levels, in patients with impaired kidney function and anemia of chronic kidney disease. The improvement of erythropoiesis by canagliflozin was independent of decreases in HbA1c, blood pressure, body weight, and other parameters in the present study.

SGLT2 inhibition shifts transport to segments further downstream of the early proximal tubule, which may exacerbate renal medullary hypoxia, although such an effect would be counteracted by reductions in blood glucose and GFR.^15^ A recent study on renal ischemia/reperfusion injury indicated that the commencement of SGLT2 inhibitor (dapagliflozin) administration at 1 day before surgery attenuated serum creatinine increases, tubular injury, and markers of apoptosis after 24 h, which were associated with increased renal levels of the transcription factor HIF-1α.^16^ Notably, this study was performed in nondiabetic mice, indicating that the renoprotective effects of SGLT2 inhibition may be independent of blood glucose control. Renal hypoxia is known to predominantly induce HIF-1α expression and upregulate the levels of the tissue protective target gene *HMOX1* (encoding heme oxygenase 1) in tubular cells.^17^ *Hmox1* mRNA expression was also increased in the kidneys of nondiabetic mice lacking SGLT2.^18^ While the effect on HIF-1α is consistent with enhancements in medullary tubular hypoxia in response to SGLT2 inhibition, it remains to be determined whether the increase of HIF-1α actually contributes to the renoprotective effects of SGLT2 inhibition. Moreover, the proximal tubule is thought to make little use of reabsorbed glucose under normal conditions, but acute kidney injury may induce a relevant glycolytic shift in the outer medullary proximal tubules,^19,20^ an effect that could involve the tubular upregulation of HIF-1α.^21^ Whether the increased delivery of glucose to this region of the kidney via SGLT2 inhibition is detrimental or beneficial in conditions of acute kidney injury remains to be determined. In comparison, the hypoxia-induced formation of EPO in peritubular interstitial fibroblast-like cells of the corticomedullary border is associated with increased levels of HIF-2α in these cells.^22,23^ The effect of SGLT2 blockade on HIF-2 remains to be determined and may provide knowledge that is relevant to the cardiovascular system.

A decrease in plasma volume, with resultant hemoconcentration, could contribute to the increase in hematocrit as observed in a previous study.^24^ However, an alternative explanation for erythropoiesis may be an effect of SGLT2 inhibition.^25,26^ In 10 diabetes patients with normal kidney function, dapagliflozin administration caused the concentration of EPO to increase until it peaked at 2–4 weeks of treatment. The reticulocyte count also increased transiently at the same time, followed by the elevation of hemoglobin and hematocrit levels.^9^ In addition, it was reported that empagliflozin treatment increased the serum EPO concentration after 4 weeks of treatment.^14^ This raises the possibility that part of the increase in the hematocrit level, which occurs rapidly and is sustained for as long as treatment continues, may be the result of enhanced erythropoiesis. The regulation of EPO production by interstitial fibroblast-like cells involves multiple factors, such as oxygen sensing through HIF, adenosine, and renin, among others.^27^ In our data, the correlation between hemoglobin and EPO levels does suggest that the release of the hormone responds to relative hypoxemia in the renal medulla. Thus, with SGLT2 inhibition, sodium escaping proximal reabsorption may impose a work overload on the distal tubule, resulting in a transient increase in oxygen consumption and a reduction in oxygen tension in the medulla.^28^ Enhanced erythropoiesis could then occur and raise the oxygen-carrying capacity of the perfusing blood, thereby re-establishing appropriate, if not better, kidney oxygenation.^29^ The present study revealed that canagliflozin administration caused the EPO concentration to increase until it peaked after 4 weeks of treatment, followed by an elevation of hemoglobin and hematocrit levels. In the present study, the level of serum ferritin was decreased, indicating that stored iron might be used for erythropoiesis due to the increase of EPO concentration.

Recent research has suggested that tubulointerstitial hypoxia is the final common pathway leading to end-stage kidney disease.^30^ It was also reported that dapagliflozin reduced oxidative stress due to excessive oxygen consumption in a mouse model of diabetic nephropathy.^31^ Furthermore, the development of progressive diabetic kidney disease has been associated with changes in proximal tubules, renal function, and prognosis and showed a higher correlation with structural lesions in the tubulointerstitium than with glomerular changes.^32^ Canagliflozin could prevent the progression of tubulointerstitial damage, since urinary L-FABP, NAG, and β_2_MG levels were reported to be reduced by canagliflozin treatment.^33^ Taken together, the increase in hematocrit during SGLT2 inhibitor therapy may indicate an improvement of hypoxia and oxidative stress in the tubulointerstitial region of the renal cortex, as well as the recovery of EPO production by interstitial fibroblast-like cells. However, the precise sequence of events starting from enhanced glycosuria to oxygen availability and stimulation of EPO production needs to be elucidated.

This study is limited by the fact that the small sample size and lack of comparator group prevent definitive conclusions from being drawn. However, the study demonstrated that a new approach using erythropoiesis parameters could clarify the distinct characteristics of patients with impaired kidney function. Future multicenter studies with large cohorts should be conducted to reassess our findings. Second, the duration of the study was short. Although the increase in hemoglobin levels was attributed to canagliflozin, we could not investigate whether this increased hemoglobin contributes to maintaining eGFR. In contrast, the improved kidney function might be attributed to the increase in EPO concentrations or hemoglobin levels. Further investigation over an extended period would be required to clarify this. Although a reduction of albuminuria was found in the short term, there was no significant change in urinary tubulointerstitial biomarkers, such as L-FABP, NAG, and β_2_MG. Further long-term study is warranted because a previous report showed that tubulointerstitial markers were reduced after 52 weeks of SGLT2 inhibitor administration.^33^

In conclusion, canagliflozin treatment leads to an improvement in erythropoiesis, including an increase of erythrocytes, hemoglobin, and hematocrit levels, in patients with impaired kidney function. The effect of canagliflozin on erythropoiesis appeared to be due to an EPO production-mediated mechanism and might be independent of glycemic control. However, because of the small sample size and short duration of this study, further studies are needed to confirm the mechanisms of erythropoiesis induced by SGLT2 inhibitors.
